# Evaluation of Population Management Based on Trap–Neuter–Return and Trap–Neuter–Adoption Practices in a Free-Roaming Cat Colony in the Federal District, Brazil

**DOI:** 10.3390/ani14172478

**Published:** 2024-08-26

**Authors:** Ana Nira Nunes Junqueira, Paula Diniz Galera

**Affiliations:** 1 Veterinary Medicine College, University of Brasília, Brasília 70.910-900, DF, Brazil; ananirajunqueira@gmail.com; 2 Brasília Environmental Institute, Brasília 70.750-543, DF, Brazil

**Keywords:** trap–neuter–return, cat management, free-roaming cats, cat overpopulation

## Abstract

**Simple Summary:**

Feline colonies cause various environmental, public health, and animal welfare problems, highlighting the importance of adequate management in promoting the One Health concept. This study evaluated the trap–neuter–return approach as a population management method for a colony of 157 animals. Following interventions conducted over 18 months, there was a 47.8% reduction in colony size, and 98.8% of the animals were sterilized. Adoption played an important role in reducing population growth. This strategy prevented kitten birth, fostered stronger bonds between caregivers and animals, and protected against death, disappearance, and abandonment. The study’s results will aid environmental and health management planning and decision-making, benefiting society, animals, and the environment.

**Abstract:**

Overpopulation of domestic animals leads to various problems, such as the formation of feline colonies. Population management methods for these colonies have been studied previously; however, no scientific consensus has been reached. This study evaluated the use of trap–neuter–return (TNR) in a free-roaming cat colony in Brazil’s Federal District. The study was conducted over 18 months and involved 157 cats that had not previously been managed. The experiment had three parts: recognition and preparation, TNR intervention, and monitoring. The results showed a 47.8% reduction in colony size. Additionally, 98.8% of the animals were sterilized. The adoption, death, disappearance, abandonment, and immigration rates were 19.7%, 14.0%, 14.0%, 7.6%, and 4.5%, respectively. The TNR experiment conducted in the proposed manner, which included detailed pre-planning, mass sterilization, active management, continuous monitoring, and educational actions, proved to be efficient and humane. However, guidelines aimed at managing animal populations, promoting adoption, preventing abandonment, and educating people about responsible pet ownership are essential for achieving sustainable results.

## 1. Introduction

Most urban centers face the problem of free-roaming animals, burdening public authorities with population control investments [1]. Although several places have implemented effective preventive actions, such as educational initiatives, registration and identification, reproduction control, and legislation related to responsible guardianship [2], many animals, especially cats, still end up as strays and form colonies. Additionally, female cats (*Felis catus*) are multiparous animals with a short gestation period of approximately 65 days and thus have the potential to produce numerous litters that can quickly reach sexual maturity [3].

These animals’ presence in public spaces directly affects the One Health concept, which integrates human, animal, and environmental health [4]. Assessing the risk they pose is important to public health, considering that approximately 60% of human diseases are zoonotic and at least 75% of the emerging pathogens that cause human infections are of animal origin [5]. Regarding environmental health, feral cats are major contributors to the predation of birds and mammals [6]. Considering the animals’ health and welfare conditions is also important as they are often neglected, and the legal provisions that protect them are frequently violated [7].

Population management methods for feline colonies have been studied and evaluated over the past few decades. These include measures such as shooting and poisoning animals, which are controversial actions that generate public outrage [8,9,10,11]. Methods involving trap–neuter–return (TNR) have been gaining popularity as non-lethal solutions. In these cases, the colonies are supported by animal rescuers and animal protection organizations, who are also responsible for carrying out the TNR. The TNR practice primarily arose from the impossibility of placing all animals in homes and shelters due to the insufficient availability of placements. Complementary actions such as vaccination, deworming, and adoption are also implemented as part of some TNR programs [12,13,14,15,16].

However, researchers have questioned the efficiency and global applicability of TNR programs, and the programs lack a strong scientific basis [7,17,18]. TNR programs’ success depends on demonstrating colony extinction or a decrease in the number of cats over time; however, few studies have provided data from an initial census with follow-up population counts [7]. Additionally, several programs that have achieved substantial and persistent cat population reductions have failed to properly document their achievements and have not publicized their success [19,20]. Furthermore, perceptions derived from modeling or other analytical exercises have been somewhat generalized and do not fully account for important local factors and contingencies [20].

In 2021, a federal law was enacted in Brazil prohibiting the elimination of stray dogs and cats, which emphasized the need for alternative methods such as TNR for colony management [21]. Literature on TNR in Brazil remains scarce, with some research having been conducted on university campuses [22,23] and at zoos [24], along with animal protection projects [25,26]. However, evidence that this approach is effective in Brazil is lacking.

This study aimed to evaluate the TNR practice as a form of population management in a free-roaming cat colony in Brazil’s Federal District.

## 2. Materials and Methods

The present study was conducted in a free-roaming feline colony in Brazil’s Federal District (183887.31, 8249434.76), which is a government area populated by office buildings and covering approximately 40,000 m^2^. The site’s perimeter was fenced or walled, and people’s access was controlled. However, cats could freely move into and out of the area. Approximately 1570 public servants worked on site. Over 18 months, the cats in the area were monitored and captured using traps to undergo the TNR protocol, which involved trapping, neutering, and returning the animals after they had recovered from anesthesia.

Before the start of the experiment, the initial colony had not been managed, and there was no veterinary care or monitoring. The animals had received irregular feeding with commercial cat food and employees’ meal leftovers.

The experiment was divided into the following three parts: recognition and preparation, TNR intervention, and monitoring.

### 2.1. Recognition and Preparation

This 3-month phase occurred prior to the TNR intervention and monitoring phases. Initially, meetings were held to formalize institutional support, establish the responsibilities of the entities involved, and obtain the necessary authorizations for animal management and for the team’s presence on site.

Once bureaucratic matters were addressed, the work began with a series of visits at different times of the day to survey the area and the cats and identify and interview the main caregivers. All gathered information was used to determine the colony’s formation history, understand the animals’ habits and temperaments, identify feeding points, estimate the initial population size, learn about human–cat coexistence rules and existing conflicts, and define educational approaches for the community.

During this phase, all the necessary materials and equipment, such as traps, transportation boxes, bait, sheets, hygiene mats, and cleaning materials, were acquired. A room at the colony site was provided to store these materials and facilitate logistics. Captured cats were also housed in the room, and the animals remained there for post-surgical recovery.

The team of handlers consisted of three individuals having experience handling and capturing cats. The handlers were up to date on rabies immunization and underwent regular antibody serological monitoring.

### 2.2. TNR Intervention

#### 2.2.1. Trapping

Animal handling was conducted over 18 months, with variations in capture frequencies depending on the availability of the veterinarian and the handlers, weather suitability, and animal behavior. The duration of each capture period, in which the traps were set, ranged from 2 h to 8 h.

Most captures occurred at twilight and nighttime when there was no staff movement and animal activity was higher. However, some individuals and kittens were captured during the daytime.

We used five cat traps (guillotine door model, height: 36 cm; width: 32 cm; length: 77 cm) and one drop trap (Fermarame^®^, Osasco, Brazil, height: 42 cm; width: 58 cm; length: 58 cm). Cat traps allow for the capture of one animal at a time and can be operated automatically or manually, whereas drop traps can capture multiple animals simultaneously and are manually operated. In some cases, a landing net (height: 140 cm, diameter: 40 cm, mesh: 2 × 2 cm) was used. The choice of trap varied depending on the assessment of individual animals’ temperaments, whether the capture was of an individual or a family, and the type of terrain (paved, grassy, or sloped). Wet cat food and canned sardines in oil were used as bait. Automatic cat traps were checked every 20 min to minimize stress and prevent excessively stressed animals from struggling.

Once captures were detected, the traps were covered with a sheet and a transportation box was attached to the trap, allowing each feline to enter the box. Occasionally, in the case of a mother with her kitten or a group of kittens, more than one animal was placed in a single box to ensure their well-being. Ten transportation boxes (height: 30 cm, width: 32 cm, length: 47 cm) were used in this experiment.

The transportation box fitted on to the trap exit, allowing for safe, effective handling. The boxes were lined with sanitary mats, covered with sheets, and labeled with the animals’ identification information and capture location to ensure that the cats could be returned to their same place of capture. The boxes containing cats were stored in the on-site room to comply with the minimum 8-hour pre-surgical fasting period. The animals were transported to the veterinary clinic using a standard vehicle.

After capture, the options for each individual animal were evaluated based on temperament, age, gestational status, and the availability of temporary foster homes or direct adoption. The following actions were taken:

1. Feral and skittish animals underwent TNR.
2. Visibly pregnant females were placed in foster homes so that their kittens could be socialized and put up for adoption. After the lactation period, mothers were either returned to the colony or offered for adoption, depending on their temperament.
3. Kittens capable of being socialized were placed in foster homes or offered for adoption.
4. Previously owned but abandoned and friendly animals were placed in foster homes or offered for adoption.

#### 2.2.2. Neutering

The sterilization procedures were performed at an accredited veterinary clinic through a government program for the population management of domestic animals. This program allowed sterilization only for animals over 6 months of age. The kittens were sterilized by private veterinary services. The animals underwent clinical evaluation and blood collection for hemograms and serum biochemistry tests (urea, creatinine, alkaline phosphatase, and alanine aminotransferase).

As previously mentioned, we decided not to neuter visibly pregnant females. When pregnancy was detected by palpation during the clinical examination, neutering was not performed, and the animal was referred to a foster home. However, females with early-stage pregnancies that were detected intraoperatively were neutered. Lactating females and those in heat were also neutered. Depending on the clinical evaluation and degree of lactation, the neuter site was accessed either ventrally or through the flank. A minimally invasive surgical technique using synthetic absorbable sutures and intradermal suturing was adopted, eliminating the need for subsequent suture removal.

During the anesthetic procedure, a V-shaped notch was made on each animal’s left ear for visual identification to prevent recapture. The animals were photographed to create a database in which all relevant information was recorded, such as origin, identification number, coat color, weight, neutering date, individual characteristics, related occurrences, and monitored sightings. All cats were immunized with the rabies vaccine.

#### 2.2.3. Return

After hospital discharge, the animals were brought back to the on-site storage room and provided with water and food ad libitum until they were fully alert. They were then released at their capture locations. The entire procedure, from capture to release, had an average duration of 24 h. After each capture cycle, all the materials were washed with a disinfectant (Herbalvet^®^, Cravinhos, São Paulo, Brazil) and the traps were checked for screw and hinge wear to prevent animal escapes and injuries.

#### 2.2.4. Adoption

Cats suitable for adoption were placed in foster homes, where they remained until permanent adoption. These temporary shelters were households that provided care and accommodation for animals in exchange for financial compensation. Some foster homes helped find adopters for the cats.

Recording each animal’s foster home entry date and adoption date allowed for the calculation of a mean time to adoption. Permanent adoptions were conducted according to responsible ownership concepts, including neutering, assessment of the adopters’ characteristics and environment, and an 8-month follow-up from the end of the experiment.

In some cases, as required by foster homes or adopters, testing for feline immunodeficiency syndrome (FIV) and feline leukemia virus (FeLV) was conducted using the Alere^®^ FIV Ac/FeLV Ag Test Kit.

### 2.3. Monitoring

Monitoring began simultaneously with capture and lasted for 18 months. During the first 4 months, efforts were focused on identifying and recognizing each animal. In this period, the number of abandonments and immigrations was considered to be zero due to the difficulty of characterizing these events among a large number of resident animals. Over the subsequent 14 months, the presence or absence of each animal was recorded in the database. Initially, sightings were recorded simultaneously with captures. As time passed and capture activities became less frequent, weekly visits with an average duration of 2 h were conducted.

For the counts, the animals were divided into adults and kittens, adults being those older than 6 months.

### 2.4. Responsible Pet Ownership Education

Educational activities were undertaken with caregivers and other staff to explain the work and its significance. Additionally, guidance was provided regarding animal health and welfare, dietary management, basic care, and animal protection legislation. Topics related to public and environmental health and their relationships with colony animals were addressed.

### 2.5. Population Dynamics

Population dynamics within the colony were evaluated both quantitatively and qualitatively over 18 months, considering entries and exits and the animals’ sex and age. The exit factors were adoption, death, and disappearance, and the entry factors were abandonment and immigration. The results are presented in a progressive timeline divided into trimesters to visualize the actions undertaken and the results obtained during the 18 months. Additionally, to assess the impact of the TNR, the presence of kittens in the colony was measured, and the condition of the colony was characterized after the experiment using information on the current number of cats and the changes observed in the feeding routine, the environment, and the relationship between people and animals. The capture effort was demonstrated by the capture attempts and the number of animals captured over time. Relevant incidents were recorded, and complication rates were measured.

### 2.6. Statistical Analysis

This was an experimental study involving a longitudinal prospective investigation in which data were collected over 18 months. Descriptive statistics were employed for the data analysis, which entailed the production of figures and tables to present the results. Microsoft Excel^®^ (https://www.microsoft.com/en-us/microsoft-365/excel, (accessed on 15 April 2024)) was used to manage the database and calculate percentages, time intervals, means, minimums, maximums, and standard deviations.

## 3. Results

Considering the total number of 157 registered animals (Table 1) and excluding 75 exits (via adoptions, deaths, and disappearances), 82 adult cats lived in the colony at the time of writing this paper, 45 (54.9%) of whom were females and 37 (45.1%) of whom were males. After 18 months of management, there was a 47.8% reduction in the colony size along with the cessation of uncontrolled reproduction among the resident animals. Of the 82 cats, only one adult male’s capture and neutering were unsuccessful, resulting in a 98.8% castration rate.

After the implementation of the educational actions, ten fixed feeding points were established, and defined time intervals for food offerings were set. Subsequent to these actions, the colony stabilized into eight sub-colonies in which the cats did not mingle but rather maintained their territory and group preferences.

The employees and caregivers reported an improved environment due to reduced cat fights and resulting injuries as well as to the cessation of cats giving birth on roofs and in rooms and the ceasing of sightings of vulnerable kittens in locations such as inside car engines and in rainy areas. Moreover, a stronger human–animal bond was observed, with the animals being given names and receiving greater care.

Of the 157 registered cats, 132 were captured in the colony, 7 were identified but died or disappeared before being captured, 1 animal did not enter any trap, and 17 were born in foster homes. Of the 132 animals captured in the colony, 89 adults (52 females and 37 males) were sterilized via a government program. Their overall mean weight was 3.18 kg (±0.87), with a female mean weight of 2.78 kg (±0.55) and a male mean weight of 3.74 kg (±0.93). Of these 52 females, 11 (21.2%) were lactating and 9 (17.3%) were pregnant.

Two females in the colony were visibly pregnant and were referred to foster homes where they gave birth to five and seven kittens each. Another female was found to be pregnant during a clinical examination and was therefore excluded from sterilization and sent to a foster home where she gave birth to five kittens. The remaining pregnant females (*n* = 6; 11.5%) underwent sterilization.

All 17 kittens born in foster homes were nursed for 45–60 days and then put up for adoption. Of the kittens’ three mothers, two were adopted after an assessment of their temperament, and one returned to the colony after sterilization.

**Table 1:** All cats registered by sex and age over the 18-month experiment.

		Sex			
		Male	Female	Indeterminate	Total
Age	Adults	43	54	-	97 (61.8%)
	Kittens	23	34	3	60 (38.2%)
	Total	66 (42.0%)	88 (56.1%)	3 (1.9%)	157

### 3.1. Exit via Adoption

**Figure 1:**
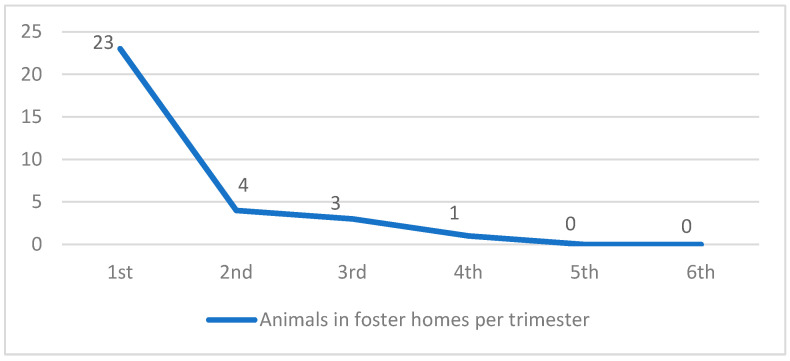
Animals in temporary foster homes per trimester.

Of the 157 registered animals, 31 (19.7%) were put up for adoption over the 18 months (Figure 1, Table 2).

The mean time until adoption was 47.9 days (in a range of 0–150 days). One animal was returned and readopted. The 8-month follow-up showed that of the 31 adopted animals, 29 (93.5%) were in good health and had adapted to their new homes, one went missing, and attempts to contact one animal’s adopter were unsuccessful. Twenty-two cats tested negative for FIV/FeLV.

### 3.2. Exit via Death

**Figure 2:**
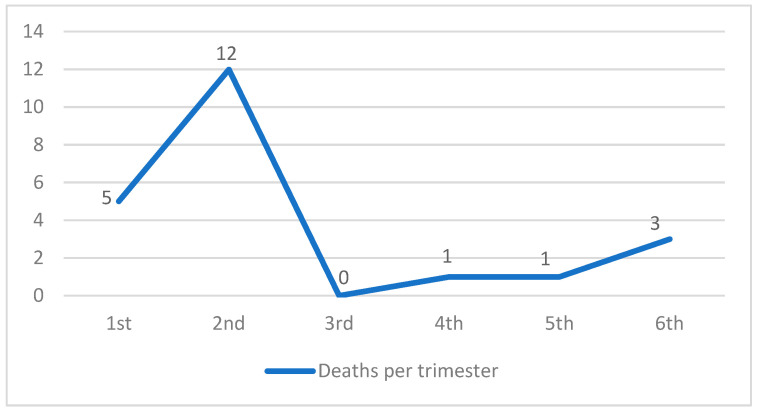
Deaths per trimester.

**Figure 3:**
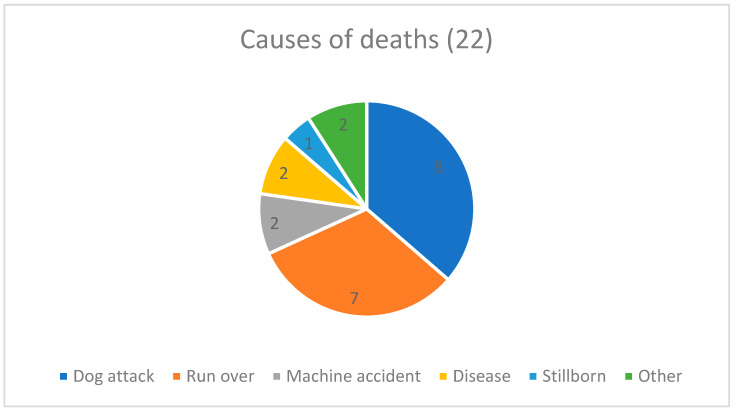
Causes of deaths. Those animals whose corpses could not undergo autopsy or which had no reports on the cause of death were categorized as “other”.

Of the 157 registered animals, 22 died (14.0%) during the 18-month experimental period (Figure 2, Table 3). Eleven were subjected to an autopsy to determine the causes of death in conjunction with caregiver reports (Figure 3).

### 3.3. Exit via Disappearance

**Figure 4:**
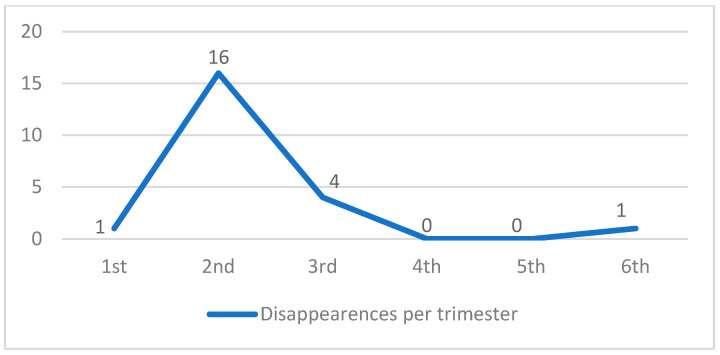
Disappearances per trimester.

Of the 157 registered animals, 22 went missing (14.0%) during the 18-month experimental period (Figure 4, Table 4). Of them, five had not yet been neutered, including three adult males. The other 17 disappeared at intervals in the range of 50–435 days after neutering.

### 3.4. Entry via Abandonment

**Figure 5:**
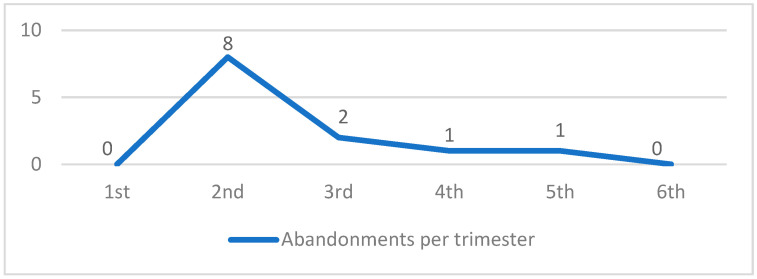
Abandoned animals per trimester.

Among the 157 registered animals, 12 (7.6%) were abandoned on site during the 18-month experimental period (Figure 5, Table 5).

### 3.5. Entry via Immigration

**Figure 6:**
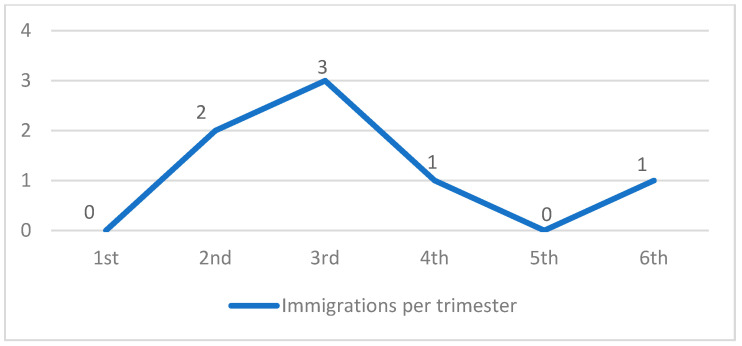
Immigrant animals per trimester.

Among the 157 registered animals, 7 (4.5%) immigrated naturally (excluding abandonment) during the 18-month experimental period (Figure 6).

### 3.6. Capture Efforts

Animal capture efforts are shown in Table 6. The mean number of animals per capture includes the days on which no cats were captured.

### 3.7. Assessment of the Presence of Kittens in the Colony

**Figure 7:**
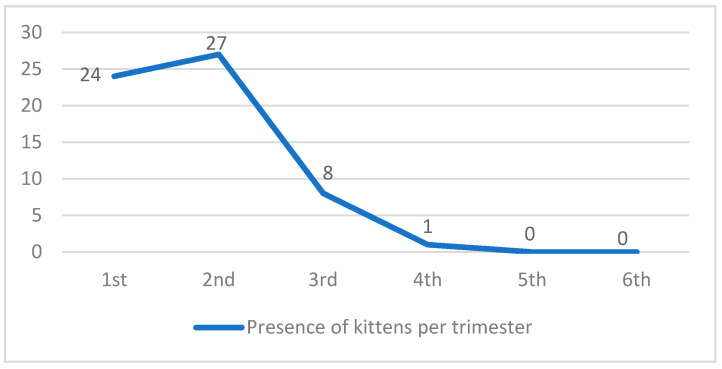
Presence of kittens per trimester.

The assessment of the presence of kittens during the 18-month experimental period is shown in Figure 7.

### 3.8. Complications

No intraoperative complications were observed. During the postoperative period, two adult female cats presented with suture dehiscence. Recapture was impossible for one of them, who had returned to the colony; therefore, no intervention was carried out, but the wound healed well by itself. The other cat was maintained in a foster home for monitoring and recovery. The postoperative complication rate was 1.3% among the 149 castrated animals.

### 3.9. Recorded Incidents

1. A cat escaped from the transportation box after capture.
2. A cat lost a claw in the trap but recovered fully without intervention.
3. Several cats presented with facial abrasions due to collisions with the trap grid.
4. An animal with ocular perforation, mandibular fracture, and traumatic palatal fissure, possibly caused by a car accident, was captured and referred for surgery. The animal recovered fully and returned to the colony.
5. Another animal received a specialized ophthalmology consultation to attend to a possibly trauma-related lesion involving the face and eyes. The animal recovered fully.
6. A handler was bitten on the forearm but did not require hospitalization.
7. A handler fell on a ramp and sustained bodily abrasions but did not require hospitalization.

## 4. Discussion

### 4.1. TNR Efficiency and Dynamics

The results indicate that the actions taken over the 18-month experimental period contained the population growth and reduced the colony size from 157 to 82 animals, representing a 47.8% decrease. Other studies have reported decline rates of 66% in 11 years [27], 78% in 9 years [15], and 85% in 23 years [28]. This project’s efficiency can be largely attributed to meticulous planning prior to trapping as well as to systematic data recording and ongoing monitoring.

The initial population estimate, according to the caregivers’ information, was approximately 30 animals. However, the number of registered animals (157) proved to be 5.2 times higher than initially estimated. This information is relevant for thorough preplanning, and the potential for caregiver underreporting must be considered. Initial uncertainty is likely to occur in any TNR program [15], and tools such as camera traps and wildlife inventory techniques can assist in establishing more accurate estimates [29].

The majority of efforts and resources were concentrated in the initial stages of management (the first and second trimesters), during which the highest number of sterilizations (72 and 28 animals, respectively) and temporary foster home placements (23 and 4 animals, respectively) was achieved. By the second trimester, 75.8% of the colony had been sterilized. Mathematical models indicate that effective population control can be achieved through high rates of continuously sterilized populations [30,31]. Sterilization should occur at a high intensity and in geographical contiguity; otherwise, reproduction, along with other factors such as animal abandonment and immigration, will theoretically sustain population growth indefinitely or until the environmental carrying capacity is reached [20,32].

The actions’ success was further reinforced by the assessment of kitten numbers, which were 24 and 27 in the first and second trimesters, respectively; however, the kitten population was reduced to zero in the fifth and sixth trimesters. Additionally, the number of abandoned animals decreased from the second to the sixth trimester until the abandoned population reached zero, demonstrating that the educational and sterilization practices contributed to the colony stabilization.

Following stabilization, the colony size is expected to decrease, with the potential for extinction; however, this will occur only when all adult animals die and if entry factors such as abandonment and immigration are controlled [20]. Spehar and Wolf reported 17 years for the extinction of a colony estimated to contain 300 animals in the United States [33].

Regarding the capture efforts (Table 6), the highest mean numbers of animals per capture (4.8 and 3.1, respectively) and the highest numbers of animals captured per attempt (10 and 8, respectively) occurred in the first and second trimesters. This is because, at the initial stage, the target population was larger, and the animals who were easier targets were captured. Thereafter, capture became more laborious owing to a decrease in the target population and the presence of more challenging animals, as Boone described [20]. Additionally, during this process, many animals may associate traps with danger through observation, odors, and noise and thus avoid them at all costs. In our experience, although we maintained the second-trimester capture efforts during the third trimester (nine attempts), the mean number of animals per capture dropped from 3.1 to 1.2.

Reference organizations for TNR have suggested strategies for optimizing trapping efforts [34,35]. Instead of exerting greater effort to capture the most challenging animals, we chose strategies to optimize this outcome, thus conserving resources and energy. In this experiment, regular feeding was suspended before trapping, different types and flavors of bait were used, the time of day when trapping was attempted varied, individual animals’ behavior was analyzed, open-trap feeding was employed to provide positive reinforcement, and the trapping attempts were temporally spaced to disassociate trapping from threats. These actions were effective in that they increased the mean number of animals captured in subsequent trimesters to 2.8, 2.0, and 3.0, respectively, across the fourth to sixth trimesters. These results demonstrate that in TNR practice, effort alone might be insufficient; employing varied, individualized strategies is necessary, often requiring reliance on handlers’ experience and knowledge.

Although this experiment involved experienced handlers using protective equipment and observing safety measures, non-serious incidents involving the people and animals were recorded. Owing to the inherent characteristics of TNR animal management, having handlers undergo training to prevent serious accidents and disease transmission is crucial.

### 4.2. Factors Influencing Exit from the Colony

#### 4.2.1. Adoptions

Of the 31 adoptions (19.7%), the majority (*n* = 27; 87.1%) were kittens. This is because cats that are not socialized in the early weeks of life can develop skittish or feral behavior [36], rendering adoption unlikely. Without adoption, this project’s population reduction rate would have been 28.0% instead of 47.8%. This corroborates Spehar and Wolf’s finding that adoption is a crucial tool in colony management because it contributes to the exit of animals and thus to higher population reduction success rates [33].

If local shelters cannot help, temporary foster homes can accommodate cats until they are permanently adopted [37]. However, this is a costly strategy because although some individuals volunteer to host animals, they must be reimbursed for the costs incurred.

In this study, the provision of FIV/FELV testing, despite burdening the program, contributed to a better chance of animals being adopted. The average time required for definitive adoption was 47.9 days, a timeline that should be considered in effective pre-planning.

The responsible adoption process requires a series of interviews with potential adopters to ensure that animals are placed in good, compatible homes and to manage adopters’ expectations and thus minimize the possibility of future abandonment [38,39]. This study had a 93.5% retention rate of the animals in adoptive homes, with follow-up conducted eight months after the end of the experiment, indicating the positive impact of the procedures used.

#### 4.2.2. Deaths

This study recorded twenty-two deaths (14.0%). The death rate decreased in the third trimester and remained stable thereafter, in line with Gunther’s observations [32]. This indicates that TNR might play a protective role. Many of the recorded deaths were due to traumatic causes (*n* = 17; 77.3%), highlighting that colony animals are exposed to several risks such as dog attacks (36.4%) and road traffic accidents (31.8%).

None of the deaths investigated through autopsy correlated with sterilization surgery, the postoperative complication rate of which was 1.3%, which is higher than that reported by Zito et al. [14], who obtained a rate of 0.29%. However, the practice, when well executed, is demonstrably safe for animals compared to other reported wound complication rates among shelter cats undergoing neutering (6.09%) and elective surgery in the general companion animal population (2.2–5.7%) [14].

Among the kittens for whom it was possible to identify the mother and her number of births, the mean number of kittens per litter was 3.6. Little reported that the complete gestational cycle of a cat lasts 4 months [3]. Considering the initial number of 54 adult females residing in the colony and assuming two annual gestations, we estimate that the actions taken prevented 388.8 births in the 12 months following the start of the study. Of those, some kittens would have died and not reached adulthood, depending on each environment’s carrying capacity (habitat, food, and predators) [20]. However, the prevention of these births demonstrates that the TNR practice is a humane way to address this issue and avoid unnecessary and traumatic deaths.

#### 4.2.3. Disappearances

During the study period, 22 disappearances were recorded (14.0%), with the highest number registered in the second trimester (16), similar to the 15% disappearance rate reported by Levy et al. [27] yet lower than the 29% reported by Swarbrick and Rand [15] and the 24% reported by Spehar and Wolf [28].

The disappearances are believed to be unrelated to neutering because in addition to the five animals that disappeared before being neutered, the intervals between neutering and the disappearances varied in the range of 50–435 days for the other 17 animals. The possible causes of disappearance are the emigration of intact males in search of females in heat, the emigration of young individuals in search of new territories, and unobserved or unreported deaths, similar to previous studies’ findings [15,28].

From the third to the sixth trimester, the number of disappearances reduced drastically, demonstrating that the TNR promoted the stabilization of the colony and that the animals settled in the territory. These outcomes were associated with improved caregiver recognition of each cat and increased care for the animals, which favored monitoring [40].

An important point to consider is that because of the factors contributing to the animals’ exit from the colony, a vacuum can occur as the local population is brought below the environment’s carrying capacity, which should be controlled through environmental and feeding management entailing proper waste disposal and control over the amount of food offered [20].

### 4.3. Factors Influencing Colony Entry

#### 4.3.1. Abandonments

Even in the areas with perimeter fencing, 24-hour surveillance, and educational initiatives, 12 abandonments were recorded over the 18 months, representing a 7.6% rate, which indicates that abandonment might occur and compromise population reduction rates [41]. The abandoned animals were mostly kittens (75.0%) and tame adults that incurred expenses related to resources and energy while occupying spaces in homes that could otherwise have been utilized for animals removed from the colony. Brazil’s laws criminalize animal abandonment [42,43]; however, accountability for abandonment is not yet a reality in Brazil.

In this context, it is essential that legislation institutionalizing community animals is approached with caution [44]. Laws regarding community animals create the notion of normalizing animals living on the streets when these measures should only be used in specific, temporary, or last-resort situations. This is because aspects related to public health, animal health, and the environment are difficult to control in colony animals [7,45,46,47]. Without actions focused on prevention and exemplary accountability for abandonment, the normalization of animals on the streets can become another incentive for this crime.

Thus, TNR actions alone might not produce the desired effect, and the implementation of population management guidelines is necessary. These may include the following [2]:

1. Situation diagnosis, including population estimation;
2. Social participation that involves various sectors in planning and execution of strategies;
3. Educational actions to promote human values, animal welfare, community health, and responsible acquisition (through purchases or adoption);
4. Environmental and waste management to reduce the sources of food and shelter;
5. Animal registration and identification;
6. Animal health care;
7. Reproductive control;
8. Prevention and control of zoonoses;
9. Animal trade control;
10. Behavioral management and proper placement of abandoned animals;
11. Legislation relevant to responsible ownership, abandonment prevention, and prevention of zoonoses;
12. Monitoring of indicators to evaluate implemented actions.

#### 4.3.2. Immigration

This study recorded seven immigrants (4.5%), all of whom were adult males. Immigration occurred at random frequencies without any identifiable correlation. This is likely due to the external pressure exerted by unmanaged areas and the search for new territories suitable for reproduction, particularly in locations with food availability [32]. As previously discussed regarding abandonment, if population management guidelines [2] are not implemented, immigration may occur frequently and compromise the objectives of TNR practices [31,41].

To mitigate these effects, contiguous areas exerting pressure should be continually evaluated, and when necessary, TNR efforts should be expanded [32]. Environmental and feeding management of animals should be considered when assessing the frequency, duration, and quantity of food offered to avoid attracting new residents and encouraging cat abandonment [20].

### 4.4. Veterinarians Trained in TNR

The government program’s 6-month age cutoff for sterilization is too restrictive for TNR practice, considering that female cats are classified as seasonally polyestrous. According to the literature, female cats enter heat at 4 months of age [48]. Given that Brazil is a tropical country with longer daylight durations, female cats can enter heat earlier.

Despite the controversies and precautions surrounding pediatric sterilization, it is necessary to raise awareness among veterinary professionals that colony animals do not have the same convenience of being sterilized as owned animals, and thus it should not be viewed as elective surgery, as established by international associations [49,50,51,52,53,54,55]. Additionally, several studies have reported no problems with prepubertal sterilization in either male or female cats [56,57,58,59].

Another issue that complicates the practice of TNR is that owing to feline physiology, a large proportion of adult females are either pregnant, lactating, or in heat. In this study, 38.5% of the female cats were pregnant or lactating (17.3% and 21.2%, respectively). Scott et al. reported an average of 19% pregnant cats, with peaks ranging from 36–47%, depending on the time of the year [60]. Wallace and Levy found an average of 15.9% pregnant cats, with peaks ranging from 36.8–58%, depending on the time of the year [61]. Cho et al. reported an average prevalence of 21.9% pregnant cats and 9.9% lactating cats [62]. If any of these states is considered an obstacle to sterilization, the practice of TNR would be impossible.

Recently, the Veterinary Medicine Council issued a regulation authorizing ear tipping as a means of identifying sterilized colony or community cats [63]; however, veterinarian resistance was encountered. We believe that this was due to a lack of knowledge about how to perform the procedure, its safety, and its importance in TNR. Even in the United States, where TNR has been practiced for several years, there is no consensus on the meaning, method, or location of ear tipping [64].

Hence, a veterinary community trained in and updated on TNR is extremely important for these programs to enable the adjustment of surgical, anesthetic, and drug protocols; the correct, safe handling of cats; and the provision of support in the case of the capture of injured animals or when euthanasia is recommended.

### 4.5. Governance and Public Policies for TNR

The Federal District has implemented actions aimed at population management of animals, such as specialized police stations for animal abuse cases and public programs for reproductive control and veterinary assistance [65,66,67]; however, various locations, such as parks, clubs, and university campuses, continue to face the problem of uncontrolled cat populations. Government data indicate that from 22 May 2017 to 1 February 2023 (5.7 years), there were 141 requests for largescale sterilization, with 47 (33.3%) requests specifically relating to colony cats [68].

The first issue to consider in the current scenario is a thorough assessment of the implementation of animal population management guidelines in the Federal District considering that these encompass varied actions and should always be based on technical and scientific grounds, with monitoring of efficiency indicators over time [2].

Second, several individuals and non-governmental organizations practice TNR informally without prior planning, data recording, or monitoring over time. Management is often voluntary, operated with limited financial resources, and performed by individuals who lack the necessary technical knowledge and a full understanding of the complexity involved [19,20]. Without detracting from the merit of these well-intentioned actions, we must emphasize that failure to meet all the required premises for a TNR program means that the effort and resources expended might not produce the desired results of reduction or extinction of the colony over time, as Natoli et al. observed [41].

The established premises do not constitute excessive bureaucratization to the point of rendering TNR impractical; instead, they are essential requirements for efficient TNR. We agree with Boone [20] that better execution can mitigate the polarized discourse surrounding the TNR method and offer an intermediate alternative.

Furthermore, isolated TNR activities have a low and temporary effect on cat colonies [32,69], rendering coordinating these events with other TNR programs and guidelines for animal population management critical. Doing so will help to avoid a vacuum caused by a decrease in the number of animals in colonies [20].

Given the inseparable interconnection amongst humans, animals, and their social and ecological environments [70], the idea that managing animals is a matter of animal welfare and public and environmental health must be normalized. Public administrations should intervene more effectively in the problem; otherwise, it will worsen in cities [18]. Even the most polarized advocates agree that inaction should not be an option [71].

Hence, establishing a government program to ensure the management, consistency, financial viability, and coordination of TNR projects with other animal-focused public policies is important. This program could be responsible for assessing environmental and health feasibility, georeferenced colony mapping, unifying information storage, prioritizing management actions, and interfacing with stakeholders.

### 4.6. Limitations

The study was conducted over 18 months and constituted the initial management of a colony for which no interventions had previously been conducted. Despite demonstrated efficiency, the duration was too short for a long-term assessment. Management and monitoring actions should be maintained, and further studies should be conducted in the coming years.

The presence of animals, even when sterilized and fed, has environmental and epidemiological impacts. Although the study site was an urban area where the impacts on native fauna were assumed to be lower and the animals were in good health at the time of the research, these factors were not evaluated. Each colony has its own configuration, leading to specificities that must be considered in each assessment, such as the type of area (urban, rural, or environmentally protected), public visits, local fauna, and the region’s epidemiological history. Thus, there are situations in which evaluation of circumstances may indicate that the TNR practice is not recommended. Such cases require alternative management solutions.

## 5. Conclusions

The TNR reduced the feline population and exerted an overall beneficial effect. However, colony extinction will only occur when the existing adult animals die and are not replaced by others, a process that takes place over the medium to long term. Adoption played an important role in reducing the population growth.

The TNR prevented the birth of kittens; fostered stronger caregiver–animal bonds; protected against death, disappearance, and abandonment; and placed animals living on the streets into good homes.

For these actions to succeed, TNR programs must be associated with other guidelines aimed at animal population management, in addition to detailed advance planning, mass sterilization on a pre-established schedule, active management, continuous monitoring, and educational actions.

## Figures and Tables

**Table 2:** Number of adopted animals by sex and age.

		Sex		
		Male	Female	Total
Age	Adults	-	4	4
	Kittens	11	16	27
	Total	11	20	31

**Table 3:** Number of deceased animals by sex and age.

		Sex			
		Male	Female	Indeterminate	Total
Age	Adults	11	4	-	15
	Kittens	3	3	1	7
	Total	14	7	1	22

**Table 4:** Number of animals that disappeared by sex and age.

		Sex			
		Male	Female	Indeterminate	Total
Age	Adults	6	9	-	15
	Kittens	1	4	2	7
	Total	7	13	2	22

**Table 5:** Number of abandoned animals by sex and age.

		Sex			
		Male	Female	Indeterminate	Total
Age	Adults	1	2	-	3
	Kittens	3	5	1	9
	Total	4	7	1	12

**Table 6:** Capture efforts undertaken in each trimester.

Trimesters	1st	2nd	3rd	4th	5th	6th	Total
Captured animals	72	28	11	14	4	3	132
Percentage	54.5	21.2	8.3	10.6	3.0	2.3	100
Accumulated percentage	54.5	75.8	84.1	94.7	97.7	100	-
Capture attempts	15	9	9	5	2	1	41
Mean no. of animals per capture	4.8	3.1	1.2	2.8	2.0	3.0	3.2
Minimum and maximum number of animals captured per attempt	0–10	0–8	0–4	1–7	1–3	3	0–10

## Data Availability

Data is contained within the article. The original contributions presented in the study are included in the article, further inquiries can be directed to the corresponding author/s.
